# Quinolone-Containing Therapies in the Eradication of *Helicobacter pylori*

**DOI:** 10.1155/2014/151543

**Published:** 2014-08-28

**Authors:** Seng-Kee Chuah, Wei-Chen Tai, Chen-Hsiang Lee, Chih-Ming Liang, Tsung-Hui Hu

**Affiliations:** ^1^Division of Hepato-Gastroenterology, Department of Internal Medicine, Kaohsiung Chang Gung Memorial Hospital and Chang Gung University College of Medicine, 123, Ta-Pei Road, Niao-Sung District, Kaohsiung 833, Taiwan; ^2^Division of Infectious Diseases, Department of Internal Medicine, Kaohsiung Chang Gung Memorial Hospital and Chang Gung University College of Medicine, 123, Ta-Pei Road, Niao-Sung District, Kaohsiung 833, Taiwan

## Abstract

Fluoroquinolones, especially levofloxacin, are used in the eradication of *Helicobacter pylori* worldwide. Many consensus guidelines recommend that the second-line rescue therapy for *H. pylori* eradication consists of a proton pump inhibitor, a quinolone, and amoxicillin as an option. Unfortunately, quinolone is well associated with a risk of developing bacterial resistance. In this paper, we review quinolone-containing *H. pylori* eradication regimens and the challenges that influence the efficacy of eradication. It is generally suggested that the use of levofloxacin should be confined to “rescue” therapy only, in order to avoid a further rapid increase in the resistance of *H. pylori* to quinolone. The impact of quinolone-containing *H. pylori* eradication regimens on public health issues such as tuberculosis treatment must always be taken into account. Exposure to quinolone is relevant to delays in diagnosing tuberculosis and the development of drug resistance. Extending the duration of treatment to 14 days improves eradication rates by >90%. Tailored therapy to detect fluoroquinolone-resistant strains can be done by culture-based and molecular methods to provide better eradication rates. Molecular methods are achieved by using a real-time polymerase chain reaction to detect the presence of a *gyrA* mutation, which is predictive of treatment failure with quinolones-containing triple therapy.

## 1. Introduction

The rate of eradication obtained using a triple therapy approach has decreased substantially for the first- and second-line regimens in recent years, owing to an increasing rate of antibiotic resistance [1]. Fluoroquinolones, especially levofloxacin, have been widely used to eradicate* Helicobacter pylori *worldwide [2]. The American College of Gastroenterology Guideline on the Management of* Helicobacter pylori *Infection [3], the second Asia Pacific consensus guidelines for* Helicobacter pylori *infection [4], and the Maastricht IV/Florence-Consensus Report [5] recommend that second-line* H. pylori* eradication rescue therapy consists of a PPI, a quinolone, and amoxicillin as an option. However, antibiotic resistance is one of the key factors responsible for failure of eradication of* H. pylori*, as well as poor compliance, high gastric acidity, a high bacterial load, and cytochrome P450 2C19 (CYP2C19) polymorphism [2, 6]. Unfortunately, quinolone is well associated with a risk of developing resistant bacterial strains [7]. Here we review fluoroquinolone-based* H. pylori *eradication regimens and discuss the challenges we are faced with owing to the emerging resistance to antibiotics that can influence the efficacy of eradication, particular the public health issue of tuberculosis.

Levofloxacin is a levorotatory isomer of ofloxacin with known activity against many Gram-negative and Gram-positive bacteria. The mode of action of levofloxacin is based on the inhibition of bacterial DNA topoisomerase II [8]. The advantage of levofloxacin-containing triple therapy is that there is an* in vivo* synergistic effect with respect to quinolone antimicrobial agents and proton-pump inhibitors (PPIs) when strains of* H. pylori *are targeted [9]. The prevalence of resistant strains is variable in different geographic areas. For example, there was zero resistance to levofloxacin in Malaysia but 8.2% resistance in Japan [10, 11]. On the other hand, increasing primary levofloxacin resistance has been reported worldwide because of plasmid-mediated horizontally transferable genes encoding quinolone resistance (18.4% in Vietnam, 20.6% in China, 63.3% in Pakistan, 29.1% in Germany, 33.9% in Portugal, 19% in Alaska, and 23% in Brazil) [12–19]. An increased use of quinolones in various different countries is probably responsible for this rise in quinolone resistance across different classes of bacteria, including* H. pylori*. Therefore, it is suggested that the use of levofloxacin should be confined to “rescue” therapy only, in order to avoid a further rapid increase in the resistance of* H. pylori* to quinolone [2]. One of our previous publications reported that quinolone therapy is effective when used to treat a susceptible infection but should be avoided when resistance is present [20].

## 2. Quinolone-Containing First-Line* H. pylori *Eradication

It is recommended that the standard triple therapy should now be avoided in areas where clarithromycin resistance is high (>15–20%) [5]. A prolonged duration to 14 days of the standard clarithromycin-based triple therapy improved the eradication rate to 82.2% but was still not good enough to attain a grade A or B report card [21]. Because of its ability to overcome metronidazole resistance, the 10-day bismuth-containing quadruple therapy could be an alternative in areas with a high prevalence of clarithromycin and metronidazole resistance but is associated with poor compliance due to side effects [22].

In sequential therapy, patients are prescribed with 5 days' dual therapy with a PPI (standard dose, b.i.d.) and amoxicillin (1000 mg, b.i.d.), followed by 5 days' triple therapy with a PPI (standard dose, b.i.d.), clarithromycin (500 mg, b.i.d.), and metronidazole (500 mg, b.i.d.) [23, 24]. At the beginning, it has been proven to be able to attain a >90% in many studies in Europe and Asia, for instance, 97% in Italian populations and 95.2% in Hong Kong [25–27], but the recent data from other countries appeared to be less effective in countries such as Korea (86.4%) and Iran (88.7%) [28, 29]. The results were even unacceptable in Latin American (76.5%) and Thailand (57.1) [21, 30].

Other alternatives include concomitant therapy and hybrid therapy, which provide >90% eradication rates even in areas with high rates of clarithromycin and metronidazole resistance. Concomitant therapy consists of a PPI (standard dose, b.i.d.) combined with clarithromycin (500 mg, b.i.d.), amoxicillin (1 g, b.i.d.), and metronidazole (500 mg, b.i.d.), prescribed all together at the same time for 7–10 days [31, 32]. It is more convenient than sequential therapy because of the shorter duration of treatment and less complex drug administration. Hybrid therapy has two phases: dual therapy with a PPI (standard dose, b.i.d.) and amoxicillin (1 g, b.i.d.) for 7 days, followed by a non-bismuth quadruple therapy consisting of a PPI (standard dose, b.i.d.), amoxicillin (1 g, b.i.d.), clarithromycin (500 mg, b.i.d.), and metronidazole (500 mg, b.i.d.) for a further 7 days. The benefit of the extended duration of amoxicillin administration is to further reduce the bacterial load to improve the eradication rate [33].

Many clinical trials chose levofloxacin in place of clarithromycin as an alternative first-line regimen. The reported eradication rates varied from 72% to 96% [34, 35]. In a recently published meta-analysis, seven trials were identified with 888 patients receiving 7 days of first-line levofloxacin and 894 treated with standard therapy (Amoxicillin, Clarithromycin and proton pump inhibitor) for 7 days. The overall crude eradication rate in the Levofloxacin group was 79.05% versus 81.4% in the standard group (risk ratio 0.97; 95% CI; 0.93, 1.02) [36]. In another meta-analysis, it was found that eradication rate in the levofloxacin-based therapy group was slightly higher than that in the standard triple therapy group regardless of treatment duration (80.2% versus 77.4%, RR = 1.03, 95% CI = 0.94–1.13) [37]. Subgroup analysis related to different geographic areas found that efficacy of 7-day standard triple regimen was statistically superior to 7-day levofloxacin-based scheme in Asian group (RR = 0.91, 95% CI = 0.86–0.97), but levofloxacin-based triple therapy was predominant regardless of treatment time in European countries (RR = 1.15, 95% CI = 1.06–1.23). It suggests that the 10-day levofloxacin-based triple therapy may be considered as an alternative for increasing cure rate of* H. pylori* infection in European areas. But in many Asian countries, standard triple regimen is still superior to levofloxacin-based therapy as first-line regimen for* H. pylori* eradication. Overall, it appeared that levofloxacin-containing triple therapy as first line regimen was not superior to standard triple therapy and both did not attain a >90% report card.

In sequential therapy the replacement of clarithromycin by levofloxacin offered an equal or better eradication rate [38], but as mentioned previously, the rapid rise in levofloxacin-resistant strains accounted for the failure of eradication. Therefore, levofloxacin-based therapy was no longer recommended as a first-line regimen.

## 3. Rescue Second-Line Quinolone-Containing Therapy 

When first-line therapy fails, the Maastricht IV Consensus Report recommends that the bismuth-containing quadruple therapy is a choice for second-line therapy [5]. However, in areas where bismuth is not available, a levofloxacin-containing triple therapy is recommended. Gisbert and De La Morena reported that a levofloxacin-containing therapy was borderline significant (81%) compared to bismuth-based quadruple therapy (70%) [39]. Extending the duration of treatment has been confirmed to improve the eradication rate. They also confirmed that the 7-day regimen was suboptimal in terms of treatment duration, and that a longer duration, for example, 10 days, might improve the eradication rate [39]. However, all the studies that used quinolone-containing triple therapy have shown that neither the 7-day nor the 10-day course is able to obtain an eradication rate >90%. Studies of 14 days' quinolone treatment were able to show an eradication rate >90% [7, 20, 40], but again, a possible increase in quinolone resistance with lengthy use is a major concern.

## 4. Rescue Third-Line Quinolone-Containing Therapy

The Maastricht IV Consensus Report recommended a selection of antibiotics for third-line regimens, depending on bacterial culture results and antimicrobial sensitivity tests [5]. A report revealed that antimicrobial sensitivity testing in patients who encountered two eradication failures showed the percentage resistance to metronidazole, clarithromycin, levofloxacin, and tetracycline to be 100%, 95%, 31%, and 5%, respectively, and they managed an eradication rate of 90% in the patients they treated by culture-guided therapy [41].

Tailored therapy according to antibiotic resistance has been proposed for achieving a high eradication rate.* H. pylori* antibiotic resistance can be classified into primary, which means there is no previous treatment for eradication of the bacterium and secondary, where a susceptible strain acquires resistance during treatment [42]. The main reasons for this phenomenon are point mutations of* H. pylori* DNA or inappropriate frequent antibiotic use [43, 44]. Tailored therapy to detect quinolone-resistant strains could offer better eradication with quinolone-containing* H. pylori* regimens. Resistances are currently detected by culture-based and molecular methods, but culture-based antibiotic sensitivity testing by *E*-test is time-consuming, and the culture rate of* H. pylori *is approximately 70–80% [45]. The same disadvantage applies to other culture-based tests, such as the agar dilution method, the breakpoint susceptibility test, and the modified disk diffusion method. Moreover,* in vitro* antimicrobial sensitivity testing does not guarantee successful eradication* in vivo*. Therefore, several attempts have been made to substitute for ineffective cultures. One of these is the use of molecular methods, such as real-time polymerase chain reaction (PCR), which can detect the existence of point mutations on quinolone resistance in* H. pylori* (N87 and D91) in the quinolone resistance-determining region of the* gyrA *gene of* H. pylori* [42]. This can be done by using gastric biopsy specimens, which can rapidly provide a >93% success rate [46–48]. The presence of a* gyrA* mutation is predictive of treatment failure with triple therapy for quinolones such as levofloxacin [45]. The advantages of this method are that there is no need for culture; the results are obtained within a few hours; it is commercially available; and it is possible to detect mutations from feces, which means that endoscopy can be avoided [48]. The disadvantages are that each mutation connected to variable antibiotic resistance needs to be determined, and that the cost may be high.

## 5. The Impact of Quinolone Exposure on Tuberculosis 

Unlike other antibiotics used as empirical treatment for community-acquired pneumonia, the quinolones have excellent activity against* Mycobacterium tuberculosis.* In a recent study, researchers found that patients recently exposed to 5 days or more of quinolone were less likely to be smear positive (OR 0.27, 95% CI 0.11 to 0.63), with an increased time to accurate tuberculosis treatment (time ratio 2.02, 95% CI 1.19 to 3.44) [49]. Furthermore, quinolone exposure for >10 days that occurred >60 days before a diagnosis of tuberculosis was associated with the highest risk of quinolone resistance (OR 17.0, 95% CI 5.1–56.8) compared to no exposure [50]. These studies highlighted the important issue that quinolone exposure is relevant to delays in diagnosing tuberculosis and the development of drug resistance. This is particularly important for doctors to bear in mind, especially among certain subsets of patients, such as those infected with human immunodeficiency virus (HIV), or in a country burdened with a high prevalence of tuberculosis such as Taiwan [51]. This could be a clinical challenge when treating* H. pylori* using an extended-duration quinolone-containing triple therapy.

There is a paucity of clinical evidence supporting the hypothesis that the use of quinolone leads to delays in treating tuberculosis in patients with* H. pylori* infection. The clinical impact of the extensive prescription of quinolones for patients with* H. pylori* infection worldwide highlighted the relationship between prior quinolone use and the subsequent emergence of quinolone resistance in* M. tuberculosis* or the delayed diagnosis of tuberculosis.

Gemifloxacin, a newer quinolone with poor activity against* M. tuberculosis *compared to levofloxacin and moxifloxacin, may be a promising alternative to overcome this problem. A dramatic increase in levofloxacin resistance after treatment failure with levofloxacin-containing triple therapy has been found in various different countries. One may need to choose a more potent quinolone in order to prevent the development of quinolone resistance during anti-*H. pylori* therapy. One recent study from Taiwan showed that gemifloxacin was superior to levofloxacin in antimicrobial activity against* H. pylori* isolates and even overcame some levofloxacin resistance [52]. Gemifloxacin is a powerful potent quinolone against* H. pylori*. It should be noted that gemifloxacin exposure is not associated with delay in tuberculosis treatment, and this has been validated in a clinical setting [53]. As a result, gemifloxacin may be the preferred quinolone for treating* H. pylori*, to alleviate any concerns about delaying tuberculosis treatment.

## 6. Conclusions

The use of quinolones such as levofloxacin should be confined to “rescue” therapy only, in order to avoid a further rapid increase in* H. pylori *resistance to quinolone. Extending the duration of treatment to 14 days has been shown to improve eradication rates, but the impact of quinolone-containing* H. pylori* eradication on public health issues such as tuberculosis treatment in such a lengthy regimen is a concern. Exposure to quinolones is relevant to delays in the diagnosis of tuberculosis and the development of drug resistance. Tailored therapy to detect quinolone-resistant strains could offer better eradication rates. This can be achieved by using culture-based or molecular-based methods such as real-time PCR to detect the presence of a* gyrA *mutation, which is predictive of treatment failure with triple therapy for quinolones, such as levofloxacin.
